# Effects of *Lentilactobacillus buchneri* and chemical additives on fermentation profile, chemical composition, and nutrient digestibility of high-moisture corn silage

**DOI:** 10.3389/fvets.2023.1296392

**Published:** 2023-12-04

**Authors:** Lei Wang, Jinze Bao, Xingliang Zhuo, Yingqi Li, Wenyuan Zhan, Yixiao Xie, Zhe Wu, Zhu Yu

**Affiliations:** ^1^College of Grassland Science and Technology, China Agricultural University, Beijing, China; ^2^State Key Laboratory of Animal Nutrition, College of Animal Science and Technology, China Agricultural University, Beijing, China; ^3^College of Animal Science, Guizhou University, Guiyang, China

**Keywords:** high-moisture corn, ensiling, additive, zein, digestibility

## Abstract

High-moisture corn silage presents a novel approach to reduce forage feeding expenses and enhance animal performance. Nevertheless, given corn’s proclivity for starch, suboptimal fermentation quality in high-moisture corn silage can lead to spoilage, posing risks to livestock well-being. Therefore, the objective of this study is to evaluate the effects of different additives on the fermentation profile, chemical composition, nutrient digestibility of high-moisture corn (HMC) silage. All treatments improved the quality of high-moisture corn silage fermentation, as demonstrated by a decrease in pH and increase in lactic acid (LA) content. The high-moisture corn silage had a low content of trans fatty acids (TFA). Fermentation effectively decreased prolamin content while increasing 48-h *in vitro* dry matter digestibility (IVDMD), estimated total tract starch digestibility (eTTSD), total digestible nutrient (TDN), and relative grain quality (RGQ) of high-moisture corn silage. Nonetheless, no effect was observed on the 48-h IVDMD of high-moisture corn silage among the different treatments. Pearson’s correlation analysis indicated that neutral detergent fiber (aNDF), neutral detergent insoluble protein (NDIP), crude protein (CP), zein, and prolamin closely correlated with the digestibility of high-moisture corn. The study’s findings demonstrate that inoculating *L. buchneri* and potassium sorbate can improve the quality of high-moisture corn silage fermentation and digestibility in different hybrids. The results will provide insights for enhancing farm productivity and profitability in China.

## 1 Introduction

High-moisture corn has served as a high-quality livestock feed, especially for ruminants and pigs, in North America and Europe more than seven decades (1, 2). Given the high latitude of the region, large-scale corn cultivation is common in Northeast China, where it can be challenging for corn to accumulate the necessary temperature for maturation (3). Consequently, corn is typically harvested in this region every October, with local farmers predominantly depending on natural drying methods to produce dry corn grains. Regrettably, this drying process frequently carries the risk of mold formation, originating from overly dense corn piles or elevated ambient humidity levels (4). To address these challenges, high-moisture corn has emerged as a new method to overcome the challenges of corn harvesting in Northeast China. It offers advantages, including earlier harvesting and decreased vulnerability to weather conditions. Consequently, high-moisture corn silage has gained popularity among dairy farms in recent years, as it is considered a vital approach to promote sustainable pasture development. This aligns with the Chinese government’s policies encouraging optimization of feed practices and environmental sustainability in the dairy industry.

Unlike other types of silage, high-moisture corn presents unique challenges during the fermentation process due to its high dry matter content (68% and above), which can make it difficult to lower the pH of the fermentation environment (5). Additionally, high-moisture corn silage is conducive to yeast reproduction, which can lead to starch loss and compromise feed quality (6). *Lentilactobacillus buchneri* was the most widely used lactic acid bacteria that can inhibit the growth of undesirable fermentation and spoilage microorganisms by producing acetic acid (7). It can also be used as a probiotic to improve animal production performance and ruminal fermentation (8). Gallo et al. (9) assessed the impact of inoculating *L. buchneri* at various silage densities and found that inoculation can enhance fermentation quality while reducing the contents of fumonisin B2 and roquefortine C. For high-moisture corn, inoculation with *L. buchneri* effectively inhibited yeast growth and reduced the relative abundance of *Enterobacteriaceae* in both the silage and under air stress conditions (10). In addition, acid/salt-based additives including acetic acid and potassium sorbate are commonly applied in silage to prevent feed spoilage (11, 12). Furthermore, silage inoculated with homofermentative lactic acid bacteria tends to have higher yeast counts due to lower acetic acid concentrations. Da Silva et al. (2) reported that using additives with potassium sorbate, sodium benzoate, and sodium nitrite as the main ingredients (at nitrite concentrations within the safe range) improved the aerobic stability and dry matter recovery of high-moisture corn silage. Hence, we believe that using additives in high-moisture corn silage preparation to inhibit deleterious microflora, improving fermentation quality and feed preservation.

Prolamins, often referred to as reserve proteins, are primarily located in the corn grain’s endosperm. Owing to their deficiency in essential amino acids alongside an abundance of non-essential amino acids, they are regarded as nutritionally inferior proteins for animal feed (13). Corn starch prolamins are encapsulated within a hydrophobic prolamin matrix, which hinders microorganisms accessibility to starch (14, 15). Previous studies (16, 17) have shown that proteolytic bacteria and plant enzymes degrading prolamins during silage improve rumen utilization and total tract starch digestibility. However, this natural proteolysis process may require up to 10 months for the protein matrix to hydrolyze to an extent that optimizes starch availability in corn silage (12). Fernandes et al. (15) observed that for different hybrids of high-moisture corn and water-rehydrated corn (RC), the hybrid type and water content did not affect the 24-h dry matter digestibility (DMD) and starch digestibility (starchD) of the same hybrid, but HMC and RC reduced the influence of vitreousness on digestibility. Therefore, selecting appropriate additives to promote the breakdown of prolamins is a crucial consideration in high-moisture corn silage preparation, in order to ensure optimal silage quality.

Therefore, this study selected two widely planted corn hybrids in Northeast China, ZN787 and LXN, to evaluate the effects of using different additives on the fermentation profile, chemical composition, and nutrient digestibility of their corn grains. The findings of this study will contribute to the advancement of high-moisture corn silage production in China and provide new dietary formulation options for pastures.

## 2 Materials and methods

Corn was sown on May 13, 2022, in the National Modern Agriculture Industrial Park of Horqin Left Middle Banner, Tongliao City, Inner Mongolia Autonomous Region, China (43°32′ ~ 44°32′N, 121°08′ ~ 123°32′E). During the planting period, seed fertilizer, nitrogen-phosphorus-potassium water-soluble fertilizer, and foliar fertilizer were applied (Whole-plant corn yield and chemical composition of the two hybrids are shown in Supplementary Table S1). Three plots of corn were randomly selected and manually harvested for each corn hybrid. The corn was manually dehusked and ground with a grinder. The grains of each corn hybrid were thoroughly mixed and randomly divided into 15 piles. Then 3 piles were randomly assigned to each treatment. Subsequently, the corn grains were subjected to different treatments: (1) Sterile deionized water (CON) (4 mL/kg) (2) Acetic acid treatment group (A4) (0.4 g/kg Fresh Weight, FW) (3) *L. buchneri* treatment group (LB) (1 × 10^6^ cfu/g) (4) Potassium sorbate treatment group (PS) (2 g/kg FW) (5) Combined treatment group of *L. buchneri* and potassium sorbate (LB + PS) (1 × 10^6^ cfu/g + 2 g/kg FW), all treatments were dissolved in sterile deionized water and sprayed evenly, the control group received the equal volume of water. Approximately 3 kg of treated corn was compacted into 3 L plastic buckets and sealed with two plastic screw top lids (inner and outer), and stored at an ambient temperature of approximately 20 ± 2°C.

After 45 days of ensiling, the high-moisture corn silage samples were retrieved from the silos, and a 25 g sample was mixed with 225 mL of sterilized distilled water and then rotated in the mixer for 2 min. Then the liquid was filtered through 4 layers of non-woven fabric and filter paper. The pH was determined by pH meter (FE28-Standard, Mettler Toledo). The filtrate was centrifuged at 10000 × g at 4°C for 5 min, and the supernatant was used to determine the Ammonia N content using the phenol-hypochlorite method (18). In addition, the supernatant was passed through a 0.22 μm filter membrane, and lactic acid, acetic acid, propionic acid, and butyric acid were determined using HPLC. The HPLC system consisted of a Shodex Rspak KC-811S-DVB gel column (30 mm × 8 mm) maintained at 50°C, an SPD-M10AVP detector, and a 3 mmol/L perchloric acid mobile phase. Detection was performed at a wavelength of 210 nm, and the injection volume was 5 μL (19).

Measurement of mean particle size (MPS) was conducted prior to silage using the People’s Republic of China national standard: Determination of feed particle size-geometric mean particle size method (GB/T 40994–2021). Raw material and silage samples were dried in a forced-air oven at 60°C for 48 h, and all dried samples were ground by a laboratory grinder and passed through a 1 mm sieve. Neutral detergent fiber and acid detergent fiber (ADF) were determined using the method of Vansoest et al. (20) with an Ankom2000 fiber analyzer. Crude protein (CP) was determined by the Kjeldahl method (21). Water-soluble carbohydrate (WSC) was determined using the method of Ke et al. (22). Ether extract (EE) and ash (Ash) were determined using the method of AOAC (23). Starch was determined by the method of McCready et al. (24). Neutral detergent insoluble protein (NDIP) was determined by the method of Licitra et al. (25). Additionally, 50 g samples were selected and dried at 55°C, and the concentrations of zein and prolamin (zein/starch) were determined according to the method of Larson and Hoffman (26). The composition of fatty acids was analyzed using gas chromatography (GC-2014 Shimadzu Corporation) with a modified method based on Qiu et al. (27). *In vitro* dry matter digestibility was performed using the Ankom RFS bottles and the pressure sensor technology described by Yuan et al. (28). Rumen fluid and buffer conditions were consistent with Xie et al. (29). Estimated total tract starch digestibility, energy and relative grain quality (RGQ) were evaluated according to the UW-Feed Grain Evaluation System (Marshfield Soil and Forage Analysis Laboratory, Hoffman and Shaver). The equations used for calculations were as follows:


$$\begin{array}{l} \mathrm{eTTSD}=(\left(99.72+\left(-0.00282*\;MPS\right)\right)+\\ \left(\left(5.97-\mathrm{Prolamin}\right)*\;\left(0.86\right)\right) \end{array}$$

$$\begin{array}{l} TDN,\%DM=(eCP+\mathrm{eStarch}+\mathrm{eNon}-\mathrm{starch}\;NFC\\ +\mathrm{eFat}+\mathrm{eNDF})-7 \end{array}$$

Where:


$$\mathrm{eCP}={CP}^{*}\;0.92$$

$$\mathrm{eStarch}={\mathrm{Starch}}^{*}\;\mathrm{eTTSD}$$

$$\mathrm{eNon}-\mathrm{starch}\;NFC={\left(NFC-\mathrm{Starch}\right)}^{*}\;0.98$$

$$\mathrm{eFat}={\left(EE-1\right)}^{*}\;2.25\;\mathrm{or}\;{\left(3.2\right)}^{*}\;2.25$$

$$\begin{array}{l} \mathrm{eNDF}={\left(NDF-\mathrm{NDIP}\right)}^{*}\;0.8\;RGQ=\left(0.223^{*}\;{\mathrm{eTTSD}}^{2}\right)\\ +\left(-34.42^{*}\;\mathrm{eTTSD}\right)+1421 \end{array}$$

Statistical analysis was performed using SPSS version 25.0. Independent sample *t*-tests were utilized to compare differences (*p* < 0.05) between the two corn hybrids raw materials. Two-way analysis of variance (ANOVA) was conducted using SPSS version 25.0 to analyze the data under the fixed effects of hybrid varieties and additives. In cases where interaction effects were significant, simple effect analyses were conducted using the GLM statement in SPSS. For data with a single fixed effect, either *t*-tests or one-way ANOVA was performed, and means were compared using the Tukey method. A significance level of *p* < 0.05 was used to determine statistically significant differences from the means.

## 3 Results

### 3.1 Chemical composition of raw materials

Table 1 shows the chemical composition of unfermented high-moisture corn. The DM and CP content were lower (*p* < 0.05) in LXN than ZN787, but aNDF, ADF, WSC, starch, EE and NDIP content were not affected (*p* > 0.05). The zein was affected by hybrid (*p* = 0.027), where LXN was 0.7 percentage-units greater compared to ZN787. Corn grains at ZN787 had a greater (*p* < 0.001) MPS than LXN.

**Table 1:** The DM content, chemical composition and *in vitro* dry matter digestibility (%DM, unless stated otherwise) of raw materials.

Item	Hybrid^1^		SEM^2^	*p*-value
	ZN787	LXN		
DM (g/kg FW)	67.38^a^	63.09^b^	1.24	<0.001
aNDF	10.38	10.91	0.22	0.075
ADF	2.83	2.91	0.09	0.534
WSC	4.72	5.26	0.31	0.557
CP	8.20^a^	7.92^b^	0.08	<0.001
Starch	70.69	70.57	0.30	0.803
EE	3.21	3.24	0.09	0.862
NDIP	0.98	1.27	0.15	0.196
Zein	5.86^b^	6.56^a^	0.25	0.027
Ash	0.79	0.79	0.04	1.000
MPS^3^, mm	3.40^a^	2.65^b^	0.22	<0.001
IVDMD	72.67	71.99	2.05	0.827

^a,b^Means in rows with different superscripts differ, *p* < 0.05.

^1^Two different hybrid corn: ZN787 and LXN.

^2^Standard error of the means.

^3^Mean particle size of unfermented high-moisture corn.

### 3.2 Fermentation profiles in high-moisture corn silage

The changes in pH and concentrations of organic acids in high-moisture corn silage during 45 days of ensiling are shown in Table 2. The interaction between H × A was evident for pH (*p* = 0.006) and lactic acid (*p* < 0.001) content. After 45 days of ZN787 high-moisture corn ensiling, A4-treated and L + P-treated ZN787 silages had lower (*p* < 0.05) pH, PS-treated ZN787 silages had greater (*p* < 0.05) lactic acid content than CON. After 45 days of LXN high-moisture corn ensiling, LB-treated LXN silages had lower (*p* < 0.05) pH. A4-treated and LP + PS-treated ZN787 silages had greater (*p* < 0.05) lactic acid content than CON. A4-treated ZN787 and LXN silages had the highest (*p* < 0.05) acetic acid content, no clear variety (*p* > 0.05) for acetic acid content was observed in other treatment groups. The propionic acid levels were similar and low among treatments. Butyric acid in all silages was not detected. Additives had different effects on fermentation profiles of high-moisture corn silages, compared with the ZN787 silages, LB decreased pH (4.44 vs. 4.23) in LXN silages (*p* < 0.05), and elevated lactic acid (0.68 vs. 1.04) and acetic acid (0.34 vs. 0.60) contents (*p* < 0.05). Therefore, additives increased fermentation quality of ZN787 and LXN high-moisture corn silage.

**Table 2:** The pH, fermentation products (%DM, unless stated otherwise) of high-moisture corn silage treated with different hybrid type and additive of ensiling.

Items^1^	Hybrid^2^	Treatment^3^					SEM^4^	*p*-value^5^		
		CON	A4	LB	PS	LB + PS		*H*	*A*	*H* × *A*
pH	ZN787	4.51^a^	4.43^bc^	4.44^abcA^	4.46^ab^	4.37^c^	0.01	0.001	<0.001	0.006
	LXN	4.44^ab^	4.39^b^	4.23^cB^	4.47^a^	4.32^b^	0.03			
LA	ZN787	0.55^bcB^	0.29^c^	0.68^bB^	0.81^b^	2.08^aA^	0.17	0.145	<0.001	<0.001
	LXN	0.99^bA^	0.42^c^	1.04^bA^	0.85^b^	1.55^aB^	0.11			
AA	ZN787	0.24^bB^	0.82^a^	0.34^bB^	0.26^b^	0.43^b^	0.06	<0.001	<0.001	0.144
	LXN	0.49^bA^	1.18^a^	0.60^aA^	0.39^b^	0.55^b^	0.08			
PA	ZN787	0.13^b^	0.20^a^	0.20^a^	0.10^b^	0.17^a^	0.01	0.668	<0.001	0.139
	LXN	0.09^b^	0.22^a^	0.22^a^	0.09^b^	0.20^a^	0.00			

^A,B, a–c^Means with different uppercase letters within a column and lowercase letters within a row differ, *p* < 0.05.

^1^BA was not detected.

^2^Two different hybrid corn: ZN787 and LXN.

^3^CON, control; A4, acetic acid at 0.4 g/kg FW; LB, *L. buchneri* at 1 × 106 cfu/g FW; PS, potassium sorbate at 2 g/kg FW; LB + PS, *L. buchneri* at 1 × 106 cfu/g + potassium sorbate at 2 g/kg FW.

^4^Standard error of the means.

^5^*H* = effect of hybrid type; *A* = effect of additives; *H* × *A* = interaction of hybrid type and additives.

### 3.3 Chemical compositions of high-moisture corn silage

As shown in Table 3, the interaction of H × A influenced contents of ADF (*p* = 0.013), zein (*p* < 0.001) and prolamin (*p* < 0.001). After 45 days of high-moisture corn ensiling, the NDF, starch and NDIP content was similar (*p* > 0.05) for both hybrids. The DM and EE content was higher (*p* < 0.05) for the ZN787 hybrid than the LXN hybrid, whereas, the zein content was lower (*p* < 0.05) for the ZN787 hybrid than the LXN hybrid. The addition of A4 decreased (*p* < 0.05) ammonia N concentrations as compared to the CON silage. The prolamin of ZN787 silage treated with L + P was lower (*p* < 0.05) than that of other treatment groups. Compared with the raw materials, the decreases in contents of NDF, NDIP and zein (*p* < 0.05, Supplementary Table S2) were observed in all treatments silage after high-moisture corn ensiling for 45 days, and the IVDMD of high-moisture corn silage was higher (*p* < 0.05, Supplementary Table S2) than that of the raw materials. No differences (*p* > 0.05) were observed in aNDF and starch from different hybrids or treatments.

**Table 3:** The DM concentration, chemical composition and *in vitro* dry matter digestibility (%DM, unless stated otherwise) of high-moisture corn silage treated with different hybrid type and additive of ensiling.

Items	Hybrid^1^	Treatment^2^					SEM^3^	*p*-value^4^		
		CON	A4	LB	PS	LB + PS		*H*	*A*	*H* × *A*
DM	ZN787	67.11^A^	67.97^A^	67.26^A^	67.87^A^	67.58^A^	0.14	<0.001	0.003	0.730
	LXN	62.39^bB^	63.56^aB^	62.24^bB^	63.19^abB^	63.26^abB^	0.17			
aNDF	ZN787	7.59	7.32	7.43	7.75	8.03	0.11	0.127	0.728	0.139
	LXN	7.20	7.60	7.55	7.40	7.32	0.08			
ADF	ZN787	2.01^b^	1.86^b^	2.00^b^	2.23^b^	3.53^aA^	0.20	0.071	0.060	0.013
	LXN	1.91	2.12	2.23	1.83	1.90^B^	0.08			
Starch	ZN787	67.44	69.66	72.00	69.99	70.43	0.49	0.937	0.613	0.366
	LXN	69.69	69.68	68.69	70.78	70.34	0.69			
CP	ZN787	8.06	7.96	8.18^A^	8.03	8.16^A^	0.03	0.045	0.144	0.320
	LXN	8.15	7.86	8.01^B^	7.49	7.89^B^	0.10			
NDIP	ZN787	0.12	0.20	0.15	0.15	0.10	0.01	0.044	0.018	0.297
	LXN	0.24	0.36	0.16	0.15	0.11	0.03			
EE	ZN787	3.57^B^	3.26^B^	3.28^B^	3.19^B^	3.43^B^	0.08	<0.001	0.335	0.901
	LXN	3.88^A^	3.82^A^	3.87^A^	3.61^A^	3.87^A^	0.05			
Ammonia N	ZN787	0.53^a^	0.21^b^	0.55^a^	0.34^ab^	0.34^ab^	0.04	0.810	<0.001	0.809
	LXN	0.52^a^	0.20^c^	0.55^a^	0.30^bc^	0.41^ab^	0.04			
Zein	ZN787	4.76^cB^	5.03^bB^	5.10^aB^	4.97^cB^	4.71^dB^	0.11	<0.001	0.048	<0.001
	LXN	6.27^bA^	5.58^cA^	6.24^bA^	6.20^bA^	6.67^aA^	0.10			
Prolamin	ZN787	7.06^aB^	7.22^aB^	7.08^aB^	7.11^aB^	6.71^bB^	0.16	<0.001	0.026	<0.001
	LXN	9.01^cA^	8.01^cA^	9.08^bA^	8.77^cA^	9.48^aA^	0.15			
IVDMD	ZN787	85.46	84.12	87.45	84.80	82.14	0.99	0.288	0.765	0.402
	LXN	85.18	83.66	83.04	83.25	83.71	0.54			

^A,B, a–c^Means with different uppercase letters within a column and lowercase letters within a row differ, *p* < 0.05.

^1^Two different hybrid corn: ZN787 and LXN.

^2^CON, control; A4, acetic acid at 0.4 g/kg FW; LB, *L. buchneri* at 1 × 106 cfu/g FW; PS, potassium sorbate at 2 g/kg FW; LB + PS, *L. buchneri* at 1 × 106 cfu/g + potassium sorbate at 2 g/kg FW.

^3^Standard error of the means.

^4^*H* = effect of hybrid type; *A* = effect of additives; *H* × *A* = interaction of hybrid type and additives.

### 3.4 Fatty acids profile of high-moisture corn silage

The fatty acids profile is listed in Table 4. The interaction of H × A influenced (*p* = 0.017) the contents of n-3. LB-treated increased (*p* < 0.05) the proportion of n-3 fatty acids in LXN silages. Linoleic acid (C18:2n6c) and oleic acid (C18:1n9c) were the two fatty acids with the highest content in high-moisture corn silage from two corn hybrids, and no differences (*p* > 0.05) were observed for them due to different additives treatment (Supplementary Table S3). In addition, low proportions of trans fatty acids were found in all treatments of ZN787 and LXN high-moisture corn silages.

**Table 4:** The fatty acids profile (of total fatty acids, unless stated otherwise) of high-moisture corn silage treated with different hybrid type and additive of ensiling.

Items^1^	Hybrid^2^	Treatment^3^					SEM^4^	*p*-value^5^		
		CON	AA	LB	PS	LB + PS		*H*	*A*	*H* × *A*
FA(g/kg)	ZN787	51.37	45.74	44.41	47.90	52.45	5.51	0.168	0.958	0.952
	LXN	44.89	33.30	43.03	34.01	33.57	3.38			
n-6	ZN787	48.45	57.42	57.07	62.15	59.28	1.95	0.138	0.244	0.225
	LXN	54.16	52.37	54.05	53.43	55.63	0.63			
n-3	ZN787	0.63^a^	0.51^abB^	0.43^bB^	0.41^b^	0.46^ab^	0.03	0.022	0.024	0.017
	LXN	0.50^bc^	0.80^aA^	0.69^abA^	0.54^bc^	0.43^c^	0.05			
n-6/n-3	ZN787	78.31	112.35	133.28	176.75	133.38	13.58	0.052	0.108	0.128
	LXN	110.36	68.86	81.15	101.91	132.61	7.34			
SFA	ZN787	22.06	19.41	18.96	20.17	19.06	0.63	0.142	0.652	0.469
	LXN	18.18	20.27	17.87	18.65	18.53	0.46			
PUFA	ZN787	49.08	57.94	57.50	62.56	59.74	1.93	0.149	0.254	0.237
	LXN	54.65	53.17	54.74	53.97	56.06	0.60			
PUFA/SFA	ZN787	2.33	3.01	3.03	3.09	3.15	0.12	0.882	0.328	0.214
	LXN	3.02	2.64	3.09	2.93	3.03	0.09			
MUFA	ZN787	28.36	22.22	23.10	24.75	20.78	1.13	0.058	0.198	0.567
	LXN	26.78	25.93	26.95	26.95	24.99	0.58			
MUFA/SFA	ZN787	1.29	1.16	1.22	1.24	1.11	0.05	0.016	0.566	0.966
	LXN	1.47	1.29	1.53	1.47	1.35	0.06			

^A,B, a–c^Means with different uppercase letters within a column and lowercase letters within a row differ, *p* < 0.05.

^1^FA, fatty acid; SFA, saturated fatty acids; MUFA, monounsaturated fatty acids.

^2^Two different hybrid corn: ZN787 and LXN.

^3^CON, control; A4, acetic acid at 0.4 g/kg FW; LB, *L. buchneri* at 1 × 10^6^ cfu/g FW; PS, potassium sorbate at 2 g/kg FW; LB + PS, *L. buchneri* at 1 × 10^6^ cfu/g + potassium sorbate at 2 g/kg FW.

^4^Standard error of the means.

^5^*H* = effect of hybrid type; *A* = effect of additives; *H* × *A* = interaction of hybrid type and additives.

### 3.5 Estimated total tract starch digestibility, summative energy and relative grain quality of high-moisture corn silage

**Figure 1:**
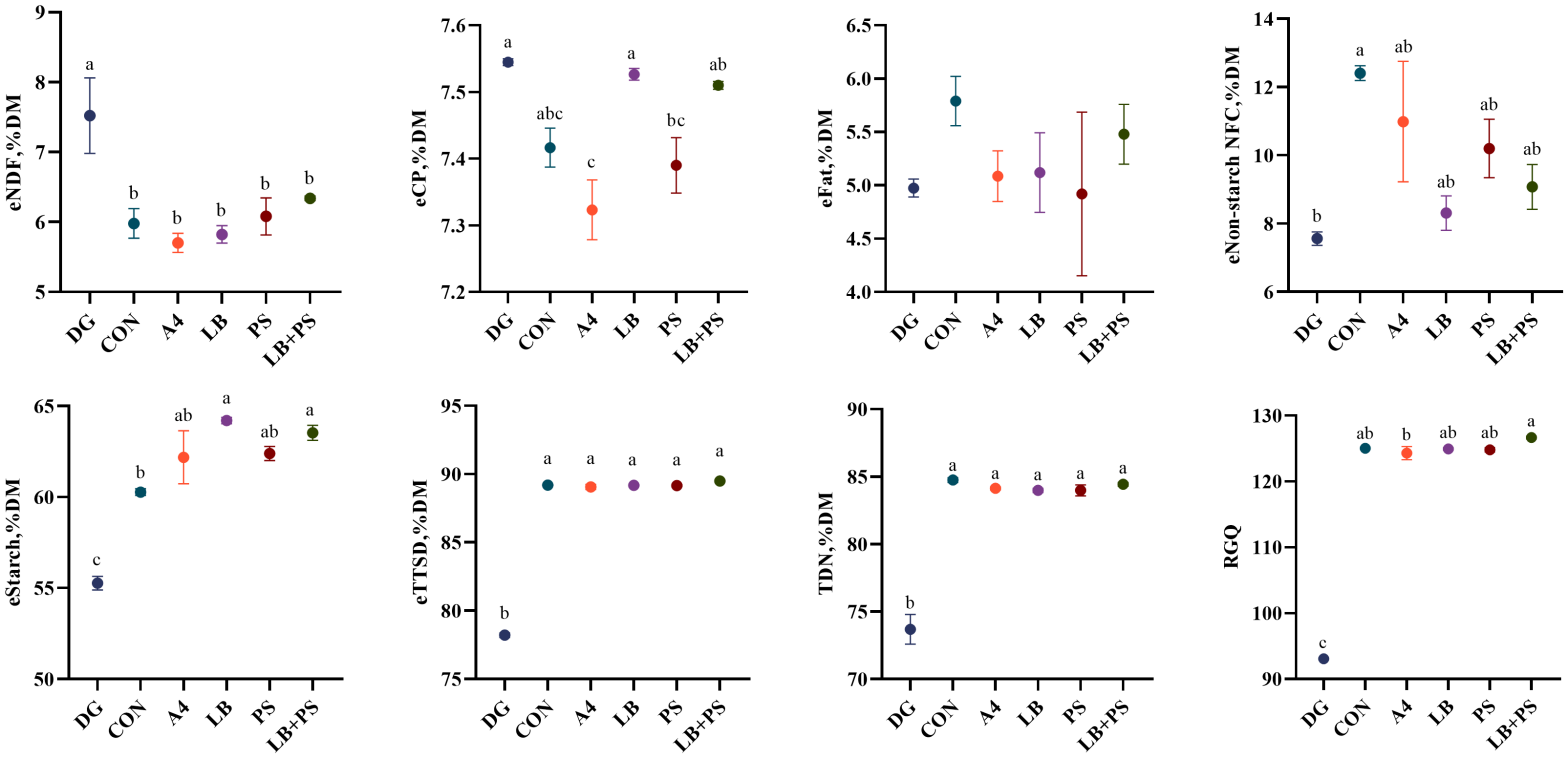
Effect of different treatments on the energy of neutral detergent fiber (eNDF), energy of crude protein (eCP), energy of fat (eFat), energy of non-fiber carbohydrate starch (eNon-starch), (eStarch), estimated total tract starch digestibility (eTTSD), total digestible nutrient (TDN) and relative grain quality (RGQ) of ZN787 high-moisture. Vertical bars are the standard errors of the means, bars with different letters differ (*p* < 0.05). DG, dry corn; CON, control; A4, acetic acid at 0.4 g/kg FW; LB, *L. buchneri* at 1 × 10^6^ cfu/g FW; PS, potassium sorbate at 2 g/kg FW; LB + PS, *L. buchneri* at 1 × 10^6^ cfu/g + potassium sorbate at 2 g/kg FW.

**Figure 2:**
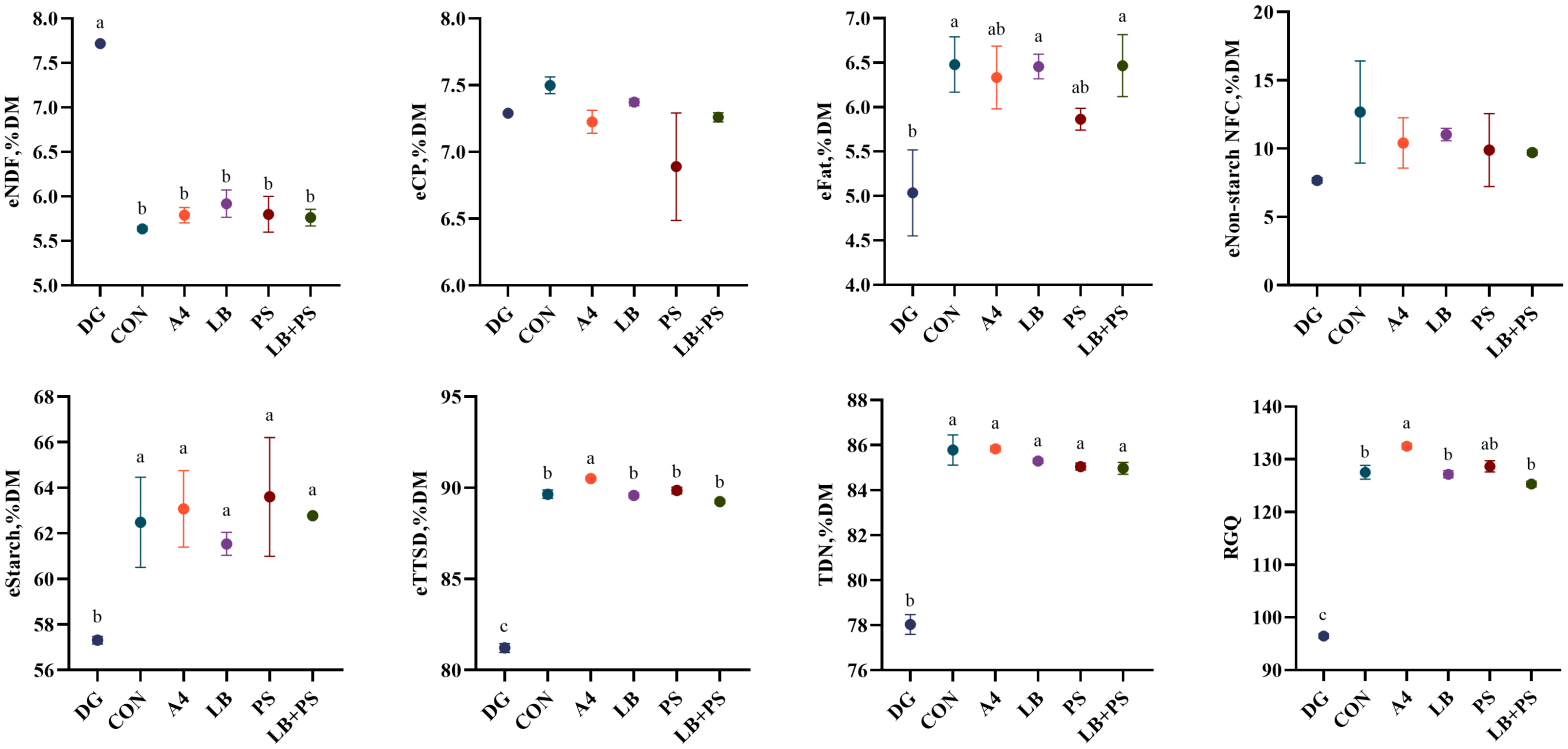
Effect of different treatments on the energy of neutral detergent fiber (eNDF), energy of crude protein (eCP), energy of fat (eFat), energy of non-fiber carbohydrate starch (eNon-starch NFC), energy of starch (eStarch), estimated total tract starch digestibility (eTTSD), total digestible nutrient (TDN) and relative grain quality (RGQ) of LXN high-moisture. Vertical bars are the standard errors of the means, bars with different letters differ (*p* < 0.05). DG, dry corn; CON, control; A4, acetic acid at 0.4 g/kg FW; LB, *L. buchneri* at 1 × 10^6^ cfu/g FW; PS, potassium sorbate at 2 g/kg FW; LB + PS, *L. buchneri* at 1 × 10^6^ cfu/g + potassium sorbate at 2 g/kg FW.

The summative energy, estimated total tract starch digestibility, total digestible nutrients and relative grain quality of HMC of the two hybrids are shown in Figures 1, 2. After 45 days of ensiling, all silages of eTTSD were higher than 89%DM, TDN higher than 83%DM and RGQ higher than 124, and LB-treated improve (*p* < 0.05) eTTSD of LXN. Both two hybrids of corn increased (*p* < 0.05) eStarch, eTTSD, TDN and RGQ compared to DG after 45 days of ensiling. The additives had similar effects on the two hybrids of high-moisture corn, and both increased (*p* > 0.05) eTTSD, TDN and RGQ. However, additives did not improve (*p* > 0.05) the energy and digestibility of high-moisture corn silage.

### 3.6 Correlation analysis of chemical components, digestibility and relative grain quality of high-moisture corn silage

**Figure 3:**
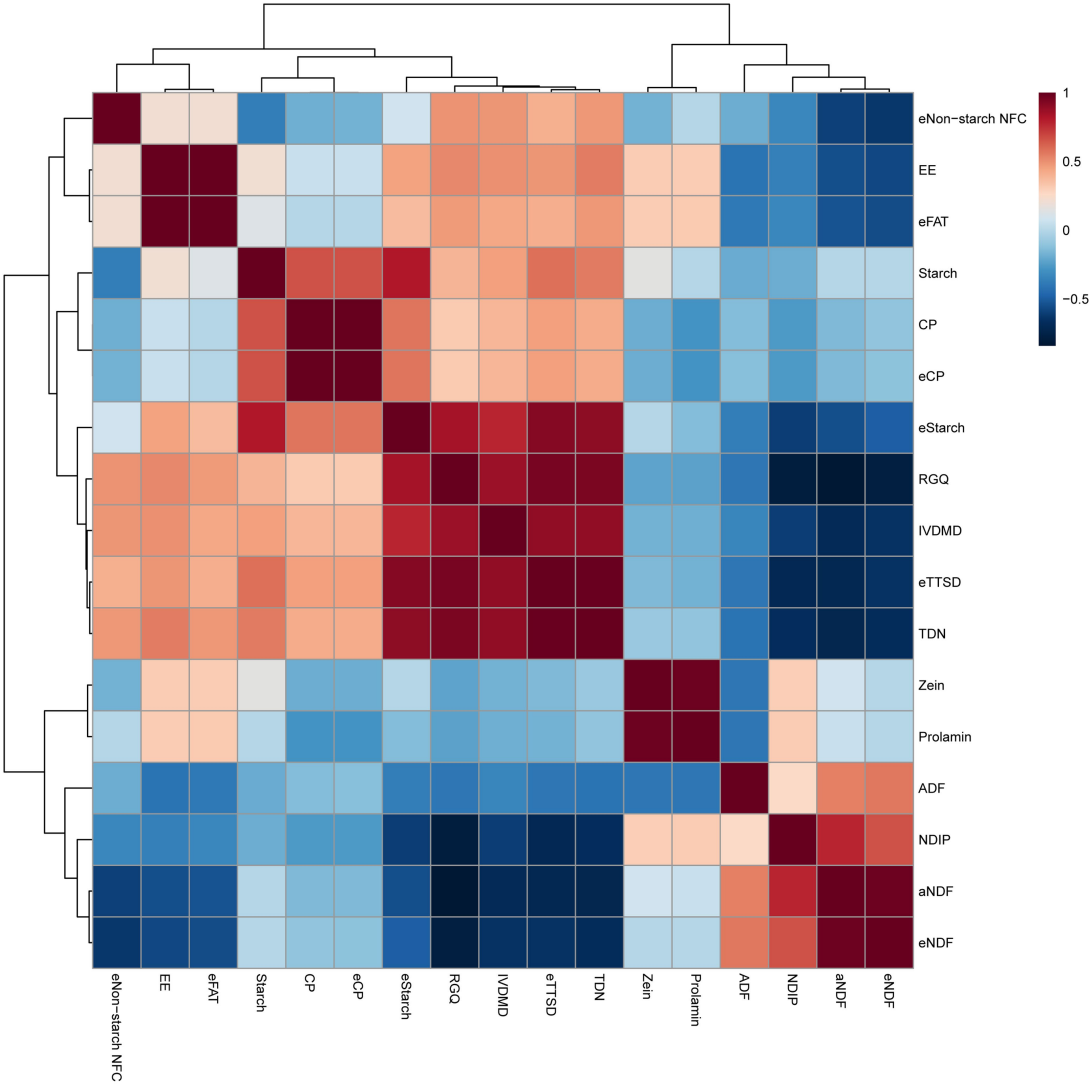
Pearson’s correlation matrix among chemical components, digestible profiles and grain quality of high-moisture corn silage. Positive correlations are displayed in red and negative correlations in blue. The correlation coefficients are proportional to numerical size and color intensity. aNDF, neutral detergent fiber; ADF, acid detergent fiber; CP, crude protein; EE, ether extract; NDIP, neutral detergent fiber crude protein; eNDF, energy of neutral detergent fiber; eFAT, energy of fat; eCP, energy of crude protein; eStarch, energy of starch; eNon-starch NFC, energy of non-fiber carbohydrate starch; eTTSD, estimated total tract starch digestibility; TDN, total digestible nutrient; RGQ, relative grain quality.

Pearson’s correlation analysis shows the correlation between multiple indicators of high-moisture corn (Figure 3). Nine chemical components related to summative energy, digestibility and relative grain quality showed positiveor negative (*p* < 0.05) correlations. Among them, NDIP correlated negatively (*p* < 0.05) with the respiratory eStarch, eTTSD, IVDMD, TDN and RGQ. Furthermore, CP correlated positively (*p* < 0.05 with eStarch, eTTSD, TDN and RGQ), and corrleated negatively (*p* < 0.05) with prolamin and zein.

## 4 Discussion

The chemical composition of ZN787 and LXN high-moisture corn hybrids was consistent with previously reported ranges (2, 15, 17). However, there were variations in the dry matter and mean particle size of ZN787 and LXN high-moisture corn at the mature stage due to hybrids differences. Mestres et al. (30) reported that high-moisture corns lead to lower MPS because it is easier to grind. Generally, endosperm type influences zein content in corn; vitreous endosperm contains abundant zein, while floury endosperm has lower zein content (31). In this study, the lower zein content in ZN787 could potentially be attributed to its higher proportion of floury endosperm. Although decreased pH and elevated lactic acid levels are widely recognized as primary indicators of adequate fermentation, pH alone does not fully evaluate silage quality. Given that high dry matter materials intrinsically demonstrate higher pH exceeding 4.2, a more holistic assessment encompassing dry matter loss, microbial composition, and other parameters is warranted (32). In contrast to the findings of Da Silva et al. (2, 33), our study on high-moisture corn hybrids deviated from the anticipated pH levels below 4.2. This discrepancy may be influenced by the type of corn endosperm or the mean particle size. In a study by Fernandes et al. (15), they investigated the fermentation process of high-moisture corn with different endosperm types and observed that the pH of high-moisture corn silage with flint hybrids exhibited a more rapid decrease and reached a lower pH after fermentation. In contrast, Saylor et al. (17) obtained high-moisture corn silage with a pH range of 3.4–3.6 by crushing the corn to smaller mean particle sizes of 1.002 and 1.046. Additives play a critical role in silage preparation, including whole-plant corn, oats, alfalfa, and others. Among the options for silage production, chemical additives and microbial additives are commonly employed. Consistent with this, the addition of the additive increased the lactic acid content in the silage compared to the control group, indicating an accelerated fermentation process for high-moisture corn. The highest lactic acid concentration was observed when inoculated with *L. buchneri* and potassium sorbate. This might be attributed to the antifungal activity of potassium sorbate against yeast, enabling the preservation of more fermentable substrates for lactic acid bacteria during the early fermentation stage. Yuan et al. (34) reported similar findings for high moisture whole-plant corn silage, as the use of natural additives limited yeast accumulation. Furthermore, potassium sorbate not only improves aerobic stability but also limits the production of ethanol, ethyl lactate, and ethyl acetate during silage fermentation (35). Its addition can improve the quality of silage with high starch content, such as high-moisture corn.

In high-moisture corn silage preparation, the development of a black layer on corn serves as a visual indicator the optimal maturity for harvesting (1). In this experiment, the two corn hybrids differed in moisture content; the LXN hybrid had an approximate moisture content of 37% during ensiling. Gomes et al. (36) reported rehydration percentages of 30, 35, and 40% for reconstituted corn grain (RCG), finding that different levels of rehydration resulted in good quality RGC, consistent with the findings of the two corn hybrids with different water content in this study. Ruminant products are a primary source of medium-chain saturated fatty acids (MCFAs) and trans fatty acids (TFAs) in the human diet. Fatty acids play a crucial role in health and associated risks. Dietary recommendations have long emphasized replacing saturated fatty acids (SFA) with polyunsaturated fatty acids (PUFA). Altering the fatty acid composition in ruminant diets can influence animal product performance, subsequently affecting human health and cardiovascular well-being (37–40). In this study, we found that high-moisture corn silage contain ample fatty acids but had relatively low levels of trans fatty acids (TFA) (C18:1n9t, C18:2n6t, Supplementary Table S3). Therefore, it can be used as a high-fatty acid diet for ruminants, helping to prevent ruminal acidosis and address energy supply limitations in ruminant animals. High-moisture corn silage had low proportions of n-3 fatty acids, and although the additives exhibited differential effects between the two hybrids during fermentation, their effect was generally small. Previous studies have shown that field wilting (41), fermentation temperature (42), and additives (43, 44) can all impact n-3 fatty acids in silage. Additionally, Li et al. (45) reported that three strains of *L. plantarum* had differential effects on the fatty acid profile of alfalfa silage. Currently, there is insufficient evidence to illustrate how additives affect n-3 fatty acids, necessitating further research. Linoleic acid (C18:2n6c) emerged as the primary fatty acid in high-moisture corn silage, capable of undergoing isomerization in the rumen to create conjugated linoleic acid (CLA). Humans cannot produce CLA and primarily obtain it through dietary intake. Feeding livestock with high-moisture corn silage can effectively increase the CLA content in red meat (beef cattle, mutton sheep) and dairy products, thereby promoting human health and sustainable dietary choices (46, 47). However, it should be noted that high-moisture corn silage has a high n-6/n-3 ratio, which may pose potential risks of inflammation. By adjusting the nutritional strategy of the diet, such as incorporating vegetable oil, oilseeds, algae, fish oil, or directly supplementing n-3 fatty acids (such as EPA and DHA), it is possible to balance the n-6/n-3 ratio and meet the dietary requirements for human health (48).

In the Cornell Net Carbohydrate and Protein System (CNCPS), protein is categorized into three components: non-protein nitrogen (PA), true protein (PB), and bound true protein (PC). PB is further divided into three parts: PB_1_, PB_2_, and PB_3_. Feeds like fermented grains and by-product feeds frequently consist a significant portion of PB_3_ components, which include prolamin protein in corn. These proteins, referred to NDIP, are insoluble in neutral washing detergents. The study a higher proportion of PB_3_ components that escaped rumen degradation was observed (49). Similar to the common silage process, the NDF of high-moisture corn in this study is also degraded, which could be one of the reasons for the reduction in NDIP content. More importantly, the decrease in NDIP indicates a reduction in the content of PB_3_ components such as prolamin. This suggests that the gliadins found in the endosperm of high-moisture corn were degraded or solubilized. The focus of research on high-moisture corn silage is its dry matter digestibility, primarily starch digestibility. Starch granules and proteins are tightly bound in corn, and the starch-protein matrix in the endosperm acts as a physicochemical barrier to ruminant starch digestion (12). In this experiment, fermentation effectively reduced the zein content in corn endosperm, which helps to increase the contact area between rumen bacteria and starch. An *in vitro* digestion test conducted for 48 h confirmed this point. Compared with the raw materials, the digestibility of the high-moisture corn increased by nearly 10%, indicating greater starch digestion and utilization, leading to the generation of more substances such as short-chain fatty acids in the rumen, providing an energy supply. In a previous study, Hoffman et al. (50) analyzed the effects of silage time and inoculation on alterations in the corn starch-protein matrix. They found that prolonged storage led to zein degradation, and that the inoculation of lactic acid bacteria had different effects on prolamin across corn hybrids. However, Junges et al. (51) and Ramirez et al. (52) believed that bacterial proteolytic activity accounted for 60% of protein dissolution. The results of this study tend to support the former conclusion. Hoffman et al. (50) experiment was inoculated with *L. plantarum*, which is commonly used in silage production. In contrast, this experiment used another common bacteria, *L. buchneri*, for inoculation. However, the test results cannot conclusively prove the contribution of silage time. The lack of noticeable degradation effect on prolamin by lactic acid bacteria observed in this study may be related to corn hybrids or silage environments. The degree of vitrification in corn endosperm varies among different hybrids, which affects the ecology of proteolytic bacteria. Additionally, the lower water content of high-moisture corn can influence bacterial activity. It is worth noting that this experiment was conducted in Northeast China in October, which might explain the difference in results from Junges et al. (51). In this study, the effect of zein degradation was investigated by adding fermentation product acetic acid and reducing the soluble protein concentration using potassium sorbate (2). The results indicated that the use of chemical additives did not yield better outcomes.

Furthermore, the UW-Feed evaluation system model was utilized to evaluate the effects of *L. buchneri* and chemical additives on corn digestibility. It was found that high-moisture corn silage remarkably improved the total tract digestibility of starch. However, it was disappointing that inoculating with *L. buchneri* or using chemical additives did not further improve corn digestibility and animal performance. Based on the strong correlation between NDIP and corn digestion and animal performance (eTTSD, IVDMD, TDN, RGQ), it is believed that zein degradation is a complex process influenced by multiple factors, including the fermentation environment, corn hybrids, chemical components, and microbial activities. Further research is needed to determine the specific contribution of bacterial action to zein degradation. Additionally, the impact of soluble proteins (globulins, albumin, prolamins, glutelin), related subunits, and amino acids on high-moisture corn silage starch digestibility remains an important topic for future research. Consequently, further research on this topic is necessary, necessitating more comprehensive experimental designs to uncover the underlying mechanisms.

## 5 Conclusion

In conclusion, high-moisture corn silage exhibited superior IVDMD, RGQ, and TDN compared with dry corn. Inoculation with *L. buchneri* and potassium sorbate proved effective in improving fermentation quality, with LXN hybrids exhibiting a higher concentration of fermentation products. This study underscored the potential of high-moisture corn silage to decrease operating costs and increase productivity for farms.

## Data availability statement

The raw data supporting the conclusions of this article will be made available by the authors, without undue reservation.

## Ethics statement

The manuscript presents research on animals that do not require ethical approval for their study.

## Author contributions

LW: Writing – original draft, Writing – review & editing, Data curation, Formal analysis, Investigation, Methodology, Supervision. JB: Writing – review & editing, Methodology, Supervision. XZ: Writing – review & editing, Formal analysis, Supervision. YL: Writing – review & editing, Investigation, Supervision. WZ: Writing – review & editing, Data curation, Investigation, Supervision. YX: Writing – review & editing, Data curation, Supervision. ZW: Writing – review & editing, Supervision. ZY: Writing – review & editing, Funding acquisition, Project administration, Supervision.
