# Supplementary material for: Effects of Lentilactobacillus buchneri and chemical additives on fermentation profile, chemical composition, and nutrient digestibility of high-moisture corn silage
Source: Front Vet Sci. 2023 Dec 4;10:1296392. doi: 10.3389/fvets.2023.1296392 (PMC10725986; doi:10.3389/fvets.2023.1296392)
Supplement: Supplementary file 1 [file Data_Sheet_1.PDF]

Supplementary Material

1 Supplementary Tables

1.1 Supplementary Table 1

Whole-plant corn yield, CP and starch content (%DM, unless stated otherwise).

Hybrid[1]	Yield (kg/ha)	CP	ST
ZN787	66000	7.09	27.80
LXN	57000	6.69	25.32

[1]Two different hybrid corn: ZN787 and LXN.

1.2 Supplementary Table 2

The chemical composition and *in vitro* dry matter digestibility (%DM, unless stated otherwise) of high-moisture corn silage and dry corn.

Items	Hybrid[1]	Treatment[2]						SEM[3]	*P*-value
		DG	CON	A4	LB	PS	LB+PB		
aNDF	ZN787	10.38[a]	7.59[b]	7.32[b]	7.43[b]	7.75[b]	8.03[b]	0.32	<0.001
	LXN	10.91[a]	7.20[b]	7.60[b]	7.55[b]	7.40[b]	7.32[b]	0.21	<0.001
NDIP	ZN787	0.98[a]	0.12[b]	0.20[b]	0.15[b]	0.15[b]	0.10[b]	0.11	<0.001
	LXN	1.27[a]	0.24[b]	0.36[b]	0.16[b]	0.15[b]	0.11[b]	0.82	<0.001
Zein	ZN787	5.86[a]	4.76[bc]	5.03[bc]	5.10[b]	4.97[bc]	4.71[c]	0.11	<0.001
	LXN	6.56[a]	6.27[a]	5.58[b]	6.24[a]	6.20[a]	6.67[a]	0.14	<0.001
IVDMD	ZN787	72.67[b]	85.46[a]	84.12[a]	87.45[a]	84.80[a]	82.14[ab]	3.23	0.009
	LXN	71.99[b]	85.18[a]	83.66[a]	83.04[a]	83.25[a]	83.71[a]	1.80	<0.001

[a-c]Means with different uppercase letters within a column and lowercase letters within a row differ, $P < 0.05$.
[1]Two different hybrid corn: ZN787 and LXN.

[2]DG, dry corn; CON, control; A4, acetic acid at 0.4 g/kg FW; LB, *L. buchneri* at 1×10[6] cfu/g FW; PS, potassium sorbate at 2 g/kg FW; LB+PS, *L. buchneri* at 1×10[6] cfu/g + potassium sorbate at 2 g/kg FW.
[3]Standard error of the means.

1.3 Supplementary Table 3

Fatty acid composition (g/kg of total fatty acid) of high-moisture corn silage after 45 days of ensiling.

Items	Hybrid[1]	Treatment[2]					SEM[3]	*P*-value[4]		
		CON	AA	LB	PS	LB+PB		H	A	H×A
C12:0	ZN787	0.21	0.25	0.19	0.16	0.21	0.02	0.666	0.599	0.942
	LXN	0.18	0.31	0.21	0.21	0.20	0.03			
C13:0	ZN787	0.14	0.17	0.14	0.13	0.16	0.02	0.982	0.817	0.966
	LXN	0.14	0.19	0.12	0.14	0.13	0.02			
C14:0	ZN787	0.08	0.02	0.07	0.05	0.05	0.01	0.069	0.499	0.687
	LXN	0.07	0.07	0.12	0.09	0.07	0.01			
C14:1n5	ZN787	0.12	0.08	0.06	0.06	0.07	0.01	0.388	0.153	0.394
	LXN	0.08	0.17	0.07	0.07	0.08	0.02			
C15:0	ZN787	0.15	0.01	0.12	0.04	0.05	0.03	0.543	0.678	0.237
	LXN	0.06	0.13	0.11	0.09	0.08	0.01			
C15:1n5	ZN787	0.05	-	0.01	0.02	0.03	0.01	0.903	0.137	0.357
	LXN	0.05	0.03	0.04	-	-	0.01			
C16:0	ZN787	18.77	16.83	16.08	17.76	16.35	0.58	0.008	0.516	0.653
	LXN	14.93	16.16	14.21	15.34	15.06	0.33			
C16:1n7c	ZN787	0.14	0.10	0.12	0.11	0.10	0.01	0.731	0.821	0.364
	LXN	0.10	0.16	0.10	0.10	0.13	0.01			
C17:0	ZN787	0.13	0.10	0.10	0.08	0.08	0.01	0.166	0.044	0.088

	LXN	0.08^{b}	0.13^{a}	0.07^{b}	0.07^{b}	0.08^{b}	0.01			
C17:1n7	ZN787	0.07	0.01	0.01	0.05	0.05	0.04	0.136	0.090	0.142
	LXN	0.05	0.02	0.03	0.01	-	0.10			
C18:0	ZN787	1.25	1.08^{B}	1.18	1.19	1.07^{B}	0.01	<0.001	0.811	0.535
	LXN	1.50	1.94^{A}	1.73	1.54	1.54^{A}	0.01			
C18:1n9t	ZN787	0.06	0.05	0.09	0.07	0.03	0.01	0.244	0.215	0.183
	LXN	0.08	0.06	0.02	0.05	0.02	0.01			
C18:1n9c	ZN787	27.81	21.85	22.71	24.33	20.37	1.13	0.062	0.236	0.593
	LXN	26.29	25.33	26.61	26.57	24.72	0.59			
C18:2n6t	ZN787	0.02	0.03	0.05	0.06	0.06	0.01	0.031	0.301	0.191
	LXN	0.05	0.13	0.11	0.05	0.05	0.01			
C18:2n6c	ZN787	47.35	56.46	56.00	61.27	58.37	1.97	0.113	0.254	0.211
	LXN	53.09	50.95	52.69	52.14	54.50	0.67			
C20:0	ZN787	0.04	-	-	0.13	-	0.03	0.577	0.577	0.434
	LXN	-	0.07	-	0.01	-	0.01			
C18:3n6	ZN787	0.46	0.38^{B}	0.44	0.27	0.31^{B}	0.03	0.007	0.203	0.274
	LXN	0.43	0.56^{A}	0.50	0.48	0.42^{A}	0.02			
C18:3n3	ZN787	0.40	0.33	0.35	0.25	0.30	0.02	0.372	0.200	0.194
	LXN	0.31	0.35	0.40	0.34	0.32	0.01			
C21:0	ZN787	1.57	1.22	1.37	0.72	1.39	0.12	0.063	0.248	0.388
	LXN	1.41	1.61	1.51	1.42	1.65	0.08			
C20:2n6c	ZN787	0.08	0.01	0.03	0.04	0.03	0.01	0.608	0.852	0.488
	LXN	0.05	-	0.12	0.03	-	0.02			
C22:0	ZN787	0.10	0.02	0.07	0.08	0.03	0.01	0.189	0.440	0.090

	LXN	0.08	0.16	0.13	0.02	0.07	0.01			
C20:3n6	ZN787	0.22	0.20	0.21	0.20	0.22	0.01	0.876	0.459	0.195
	LXN	0.19	0.29	0.22	0.20	0.16	0.02			
C22:1n9	ZN787	0.06	0.10	0.04	0.05	0.08	0.01	0.196	0.428	0.168
	LXN	0.08	0.09	0.08	0.13	0.04	0.01			
C20:3n3	ZN787	0.03	0.01	-	0.02	0.01	0.01	0.135	0.371	0.142
	LXN	0.02	0.07	0.04	-	-	0.02			
C24:0	ZN787	0.11	0.15	0.08	0.16	0.09	0.01	0.612	0.013	0.944
	LXN	0.10	0.12	0.09	0.16	0.08	0.01			
C20:5n3	ZN787	0.11	0.15	0.08^{B}	0.16	0.09	0.02	0.023	0.058	0.078
	LXN	0.10	0.12	0.09^{A}	0.16	0.08	0.02			
C24:1n9	ZN787	0.05	0.03	0.06	0.06	0.07	0.01	0.090	0.732	0.117
	LXN	0.05	0.08	-	0.03	-	0.01			
C22:6n3	ZN787	0.02	0.04	-	0.06	0.07	0.01	0.451	0.528	0.960
	LXN	0.05	0.09	0.11	-	-	0.02			

[A,B, a-c]Means with different uppercase letters within a column and lowercase letters within a row differ, $P < 0.05$.

[1]Two different hybrid corn: ZN787 and LXN.

[2]CON, control; A4, acetic acid at 0.4 g/kg FW; LB, *L. buchneri* at 1×10^6 cfu/g FW; PS, potassium sorbate at 2 g/kg FW; LB+PS, *L. buchneri* at 1×10^6 cfu/g + potassium sorbate at 2 g/kg FW.

[3]Standard error of the means.

[4]H = effect of hybrid type; A= effect of additives; H×A = interaction of hybrid type and additives.
